# Choroid Plexus Abnormalities in Autoimmune Encephalitis

**DOI:** 10.1002/cns.71048

**Published:** 2026-09-10

**Authors:** Yueqian Sun, Ning Wang, Shihao Ge, Li Feng, Qun Wang

**Affiliations:** ^1^ Department of Neurology, Beijing Tiantan Hospital Capital Medical University Beijing China; ^2^ National Center for Clinical Medicine of Neurological Diseases Beijing China; ^3^ Department of Neurology, Xiangya Hospital Central South University Changsha China; ^4^ National Clinical Research Center for Geriatric Disorders Xiangya Hospital, Central South University Changsha China; ^5^ Department of Neurology The First Affiliated Hospital of Zhengzhou University Zhengzhou China

**Keywords:** autoimmune encephalitis, autoimmune encephalitis‐associated epilepsy, choroid plexus, magnetic resonance imaging, prognosis

## Abstract

**Background:**

Recent evidence suggests that the choroid plexus (ChP) serves as a gateway for neuroinflammation. Autoimmune encephalitis (AE) features a high inflammatory burden, yet the role of ChP in AE remains unclear. This study compared choroid plexus volume (CPV) at symptom onset between AE patients and healthy controls (HCs), and evaluated its association with prognosis.

**Methods:**

This single‐center study included 151 AE patients with definite autoantibodies. ChP volume (CPV) was segmented from baseline 3D‐T1 MRI and normalized. Baseline CPV was compared between AE and age‐ and sex‐matched HCs, and its associations with 12‐month functional outcomes (mRS) and progression to autoimmune encephalitis‐associated epilepsy (AEAE) were assessed. A logistic regression model based on ChP radiomic features was developed to predict AEAE. Exploratory cerebrospinal fluid (CSF) proteomics and metabolomics were performed in a small subset to generate hypotheses on underlying pathways.

**Results:**

Of 151 AE patients, 141 completed 12‐month follow‐up; 26 (18.4%) developed AEAE. AE patients had significantly larger CPV than controls (1.235 ± 0.274 vs. 1.035 ± 0.264, *p* < 0.001). Larger baseline CPV was significantly associated with AEAE and mRS (*p* < 0.05). A two‐feature radiomic model (RunEntropy and original volume) predicted AEAE with AUCs of 0.766 (training) and 0.851 (test). Exploratory proteomics in a subset suggested CPV‐correlated downregulation of glutamatergic synapse pathways and upregulation of PI3K‐Akt/integrin pathways.

**Conclusions:**

Baseline CPV was greater in AE patients than in HCs, and larger baseline CPV was associated with progression to AEAE and poor one‐year functional outcomes. ChP imaging may aid in early risk stratification for AE patients.

## Graphical abstract

Choroid plexus (ChP) enlargement is a robust AE imaging biomarker, with independent prognostic value for AEAE and poor outcomes. ChP MRI radiomic model predicts AEAE well, leveraging shape features to provide effective predictive performance for AEAE. Multi‑omics links ChP enlargement to reduced glutamatergic synapse pathway activity and enhanced PI3K‑Akt/integrin signalling, revealing BCSFB dysfunction mechanisms.

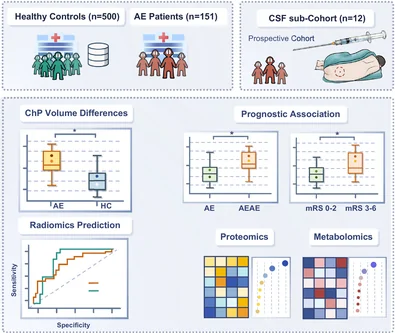

## 1 Introduction

The choroid plexus (ChP) is a highly vascular tissue situated within the four cerebral ventricles, responsible for cerebrospinal fluid (CSF) secretion [1]. It forms the blood‐CSF barrier (BCSFB), acting as a boundary between blood and CSF [1, 2, 3]. Beyond CSF production, the ChP influences neurogenesis, neuroprotection, and waste clearance [1, 2, 4, 5, 6]. Studies in multiple sclerosis (MS) have shown that ChP structural changes correlate with central nervous system (CNS) immune cell infiltration [3, 6, 7], and similar findings have been reported in Alzheimer's disease (AD), suggesting that ChP enlargement may serve as a marker of neuroinflammation across various neurological disorders.

Autoimmune encephalitis (AE) encompasses a heterogeneous group of disorders characterized by CNS inflammation and subacute clinical symptoms, with seizures as a presenting or core clinical feature [8, 9]. Animal and human studies suggest that immune cell entry into the CNS via meningeal lymphatics and blood–brain barrier (BBB) disruption drives AE pathogenesis, where cytokine/chemokine‐induced BBB leakage allows autoantibodies and immune cells to infiltrate brain parenchyma [10, 11, 12, 13, 14, 15]. Despite generally favorable functional recovery following immunotherapy—and effective seizure control with initial immunotherapy and/or antiepileptic treatment in most patients—clinical observations reveal substantial heterogeneity in prognosis. A subset of patients progress to chronic neurological deficits, including drug‐resistant epilepsy [16]. In response to this heterogeneity, the International League Against Epilepsy (ILAE) Autoimmunity/Inflammation Task Force recently revised the terminology to “autoimmune encephalitis‐associated epilepsy” (AEAE), defining epilepsy secondary to AE regardless of surface or intracellular antibody associations [17]. This distinction carries critical therapeutic implications: acute symptomatic seizures secondary to AE are primarily managed with immunotherapy, whereas seizures in AEAE require targeted antiseizure medications (ASMs), as immunotherapy typically yields only partial improvement and complete recovery or seizure freedom remains rare [18]. Accordingly, these divergent outcomes also have distinct social consequences, underscoring the pressing need for prognostic assessment.

Given the ChP's established role as a brain‐immune interface and its involvement in other neuroinflammatory disorders, it may be similarly implicated in AE pathogenesis. Recent studies show that peripheral blood mononuclear cells (PBMCs) from AE patients upregulate IL‐1β, inhibiting BBB tight junction proteins and promoting autoantibody/immune cell infiltration into brain parenchyma [19]. The ChP also produces and expresses receptors for IL‐1β, TNF‐α, and IL‐6, playing a role in peripheral and central immune responses [20, 21]. As a brain‐immune interface, ChP volume changes may affect CSF autoantibody clearance efficiency. However, research on ChP and ventricular structural/functional changes in AE remains limited, and no study has systematically investigated ChP volume alterations in this population.

Therefore, this study focused specifically on baseline ChP changes in AE. We aimed to: (1) confirm ChP enlargement at AE onset; (2) assess whether baseline ChP volume correlates with long‐term prognosis and the development of AEAE; and (3) develop and validate a ChP radiomics‐based model for predicting AEAE. As an exploratory complement, we performed CSF proteomic and metabolomic analyses in a small patient subset to generate hypotheses on potential mechanisms underlying ChP changes.

## 2 Methods

### 2.1 Participants and Clinical Data

This study enrolled patients from the Epilepsy Department of Beijing Tiantan Hospital between January 2018 and June 2024, with a total of 151 patients included. Inclusion criteria were: age 16–85 years; diagnosis of definite AE according to the Graus et al. 2016 criteria [8], with well‐characterized neuronal surface or synaptic autoantibodies detected in serum and/or CSF by cell‐based assays (CBAs; Euroimmun, Lübeck, Germany); availability of a 3T brain MRI scan obtained before immunotherapy as baseline MRI. Exclusion criteria were: seronegative AE (probable or possible AE based on clinical and paraclinical features but without detectable antibodies); a premorbid Modified Rankin Scale (mRS) score > 0; clinical or subclinical seizures within 2 h prior to MRI scanning, or MRI motion artifacts; a history or MRI evidence of other neurological disorders known to affect choroid plexus volume (CPV), including MS, neuromyelitis optica spectrum disorder, AD, idiopathic intracranial hypertension, or central nervous system infection. All participants provided written informed consent for the use of their medical records, and the study was conducted in accordance with the Declaration of Helsinki.

All 151 patients underwent MRI‐based ChP analysis. Follow‐up data, including mRS scores and seizure status, were recorded for each participant. Additionally, CSF samples were prospectively collected from a subset of 12 patients for subsequent proteomic and metabolomic analyses.

**FIGURE 1:**
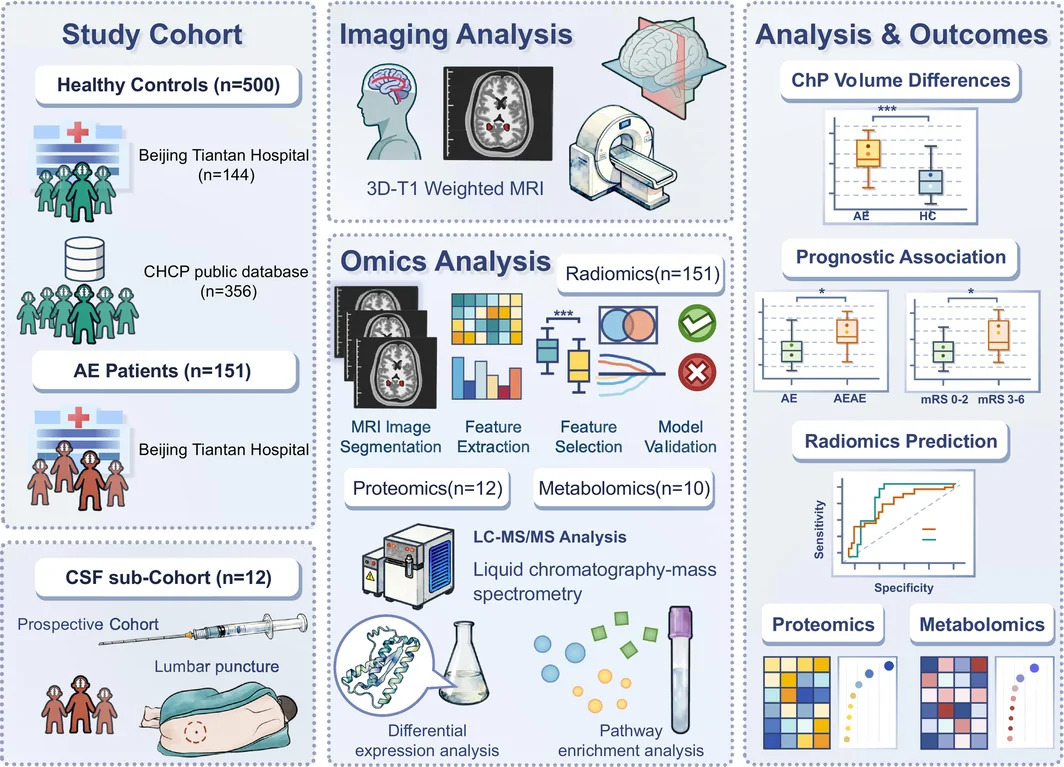
Study overview.

A total of 500 age‐ and sex‐matched healthy controls were enrolled from two sources: 144 from Beijing Tiantan Hospital and 356 from the Chinese Human Connectome Project (CHCP) public database [22]. Of the 362 initially available CHCP participants, six were excluded due to failed FreeSurfer processing. All HCs were free of neuropsychiatric disorders (Figure 1).

### 2.2 Choroid Plexus Volume Analysis

#### 2.2.1 Brain Structural Neuroimaging Acquisition

Structural three‐dimensional T1‐weighted imaging (3D‐T1WI, voxel size: 1.0 × 1.0 × 1.0 mm^3^) was performed using a 3.0‐T MRI scanner (SIGNA Premier; GE Healthcare, Milwaukee, WI, USA) with a 48‐channel head coil. Scan parameters for 3D‐T1WI were: repetition time 7.3 ms, echo time 3.0 ms, inversion time 450 ms, flip angle 12°, field of view 256 mm × 256 mm, acquisition matrix 256 × 256, slice thickness 1.0 mm, 176 slices, and scan time 4 min 56 s.

#### 2.2.2 Image Segmentation and Volume Calculation

FreeSurfer 7.2 [23, 24] was used to automatically segment gray matter (hippocampus, HIP), lateral ventricular volume (LVV), and estimated total intracranial volume (eTIV) from 3D‐T1WI sequences. All regional volumes were normalized to eTIV to eliminate confounding effects of head size and ventricular expansion.

The ChP was segmented from 3D‐T1WI using a 3D nnU‐Net deep learning algorithm [25]. ChP imaging was quality‐checked by a neuroradiologist, which served as ground truth for model training. A total of 165 cases with expert annotations were used for model training via five‐fold cross‐validation (150 epochs; mean Dice score of 0.861). An independent set of 72 cases was used as the test. The trained model was then applied to all study images for automated ChP segmentation and subsequent volume calculation. Manual corrections using ITK‐SNAP were implemented for segmentations extending beyond ventricular boundaries, incorporating non‐ChP tissues, or exhibiting abnormal 3D morphology. For FreeSurfer brain tissue segmentation QC, initial inaccuracies were identified via Euler numbers [26], followed by visual validation and corrections using FSLeyes per official FreeSurfer guidelines (https://surfer.nmr.mgh.harvard.edu/fswiki).

#### 2.2.3 Volume‐Based Statistical Analysis

We compared CPV between AE and HC groups to assess ChP enlargement in AE, and examined the correlation of baseline CPV with mRS and AEAE development. Receiver operating characteristic (ROC) analysis with the Youden index was used to determine the optimal cutoff of normalized CPV for predicting AEAE; area under the curve (AUC), sensitivity, specificity, and 95% CIs were calculated. Additionally, a bootstrapped mediation model (5000 iterations) tested whether HIP volume mediated the relationship between CPV and AEAE development. The total effect was decomposed into direct (ChP → AEAE) and indirect (through HIP volume) effects, with mediation effect size reported as NIE/TE × 100 [27].

### 2.3 Radiomics Analysis

#### 2.3.1 Feature Extraction and Selection

From the ChP segmentation mask on T1WI, a total of 944 quantitative radiomic features were automatically extracted and categorized into four major classes: (I) intensity features; (II) shape and size features; (III) texture features; and (IV) wavelet features.

First, the data were randomly split into training and validation sets at a 7:3 ratio. To reduce redundancy and noise in the high‐dimensional data and enhance generalizability, a two‐stage feature selection strategy was applied to the training set. First, the intraclass correlation coefficient (ICC) was calculated for each feature, retaining only those with high stability (ICC > 0.8) to minimize interobserver variation. Second, the remaining stable features were tested using the independent samples *t*‐test or Mann–Whitney *U* test; features failing either test were excluded. Additionally, LASSO was applied to the training set to select features with non‐zero coefficients that can differentiate patients with chronic epilepsy from those without. All calculations followed the PyRadiomics 2.2.0 documentation [28].

#### 2.3.2 Model Development and Validation

The logistic regression model was trained and evaluated on the test set using selected features. The model's predicted probability was defined as the radiomics signature. Performance (AUC, sensitivity, specificity) was assessed on the training set and validated on the test set via ROC curves. Calibration curves and the Hosmer–Lemeshow test were used to evaluate agreement between predicted and observed chronic epilepsy status. Decision curve analysis (DCA) [29] quantified the net benefit across threshold probabilities to assess clinical utility.

### 2.4 Proteomics and Metabolomics Analysis

#### 2.4.1 Proteomics Analysis

CSF proteomics was performed using a 4D‐DIA strategy on a U3000 HPLC‐Orbitrap Exploris 480 system. Peptides were separated on a C18 column over a 45‐min gradient (7%–30% B) with buffers A (98% H_2_O, 2% ACN, 0.1% FA) and B (98% ACN, 2% H_2_O, 0.1% FA). DDA was used for library generation, followed by DIA acquisition with FAIMS Pro. Data were analyzed in Proteome Discoverer v2.4 against the UniProtKB human database (April 18, 2022) with 10 ppm precursor and 0.02 Da product ion tolerances, filtered to 1% FDR. After quality control, 3633 proteins remained. Pearson's correlation with normalized CPV identified a total of 204 proteins with |*r*| > 0.4 and FDR‐adjusted *p* < 0.05 as CPV‐correlated proteins. Gene enrichment analysis characterized biological processes (BPs) in this protein set. For blood–CSF barrier association, choroid plexus expression was assessed using Human Protein Atlas consensus RNA data (nTPM ≥ 1). High‐confidence physical interactions (combined score ≥ 0.7) between the proteins and barrier core proteins (TJP1, CDH1, PARD3, LAMB1) were evaluated using STRING. KEGG pathway and Disease Ontology analyses were performed; adjusted *p* < 0.05 was considered significant [30, 31].

#### 2.4.2 Metabolomics Analysis

CSF metabolomics was performed on a Vanquish UHPLC coupled to a Q Exactive HF‐X in positive and negative modes. Samples were separated on a Hypersil Gold column (100 × 2.1 mm, 1.9 μm) over a 17‐min gradient at 0.2 mL/min. Positive mode used 0.1% formic acid vs. methanol; negative mode used 5 mM ammonium acetate (pH 9.0) vs. methanol. Raw data were processed in Compound Discoverer 3.1. Peaks were matched against mzCloud, mzVault, and MassList, and metabolites were annotated using KEGG. After preprocessing, 2733 metabolites remained and were log_2_‐transformed. Pearson's correlation of metabolites versus normalized CPV identified 956 metabolites with |*r*| > 0.4 (*p* < 0.05) as CPV‐correlated. Subsequent gene enrichment analysis characterized enriched BPs, and KEGG pathway enrichment analysis was performed for these metabolites [30, 31].

#### 2.4.3 Integrative Analysis of Proteomics and Metabolomics

For integrated analysis, Spearman correlation analysis (|correlation coefficient| > 0.5) built a proteomics‐metabolomics correlation network. A machine learning workflow was used: a random forest model (mean decrease accuracy/gini for variable importance) screened multi‐omics biomarkers, logistic regression adjusted for imbalanced clinical parameters, and 10‐fold cross‐validated AUC assessed predictive performance.

### 2.5 Statistical Analysis

Categorical variables are presented as numbers (percentages) and analyzed using the *χ* ^2^ test. Normally distributed continuous variables are expressed as mean (SD) and evaluated by ANOVA or independent *t*‐test; skewed data are reported as median (IQR) and assessed by Kruskal–Wallis or Mann–Whitney *U* test. Correlations were performed using Pearson's coefficient. Unless otherwise specified, two‐tailed *p* < 0.05 was considered statistically significant. For the proteomics analysis involving multiple comparisons, significance was defined as FDR‐adjusted *p* < 0.05. All statistical analyses were performed using SPSS version 29.0 (SPSS Inc., Chicago, IL, USA).

## 3 Results

A total of 151 AE patients (mean age 46.47 ± 18.73 years, 54.3% male) and 500 age‐ and sex‐matched HCs (43.09 ± 22.41 years, 53.6% male) were enrolled. The AE subgroup distribution was as follows: 44 with NMDAR encephalitis, 78 with LGI1 encephalitis, 19 with GABAB receptor encephalitis, and 11 with GAD65‐related encephalitis. eTIV was comparable between groups (AE: 1,481,343.8 ± 176,973.5 mm^3^; HC: 1,497,878.1 ± 183,564.3 mm^3^; *p* = 0.328). Table 1 summarizes the main demographic, clinical, and volumetric data of the AE patients.

**TABLE 1:** Demographic characteristics in patients with autoimmune encephalitis.

Characteristics	
Demographics	
Gender (male)	82 (54.3%)
Age, y	46.47 ± 18.73
Duration from onset to first MRI,[day, M (P25, P75)]	46 (20, 152.5)
Subtypes (*n*, %)	
LGI1‐AE	78 (51.66%)
NMDAR‐AE	44 (29.14%)
GABAB‐AE	18 (11.92%)
GAD65‐AE	11 (7.28%)
Clinical features	
Prodromal symptoms	64 (42.4%)
Cognitive impairment	124 (82.1%)
Behavioral and mood disturbances	105 (69.5%)
Seizure	138 (91.4%)
Status epilepticus	25 (16.6%)
Sleep disorders	90 (59.6%)
Abnormal EEG	88 (58.3%)
Abnormal MRI	109 (72.2%)
Immunotherapy	
First‐line immunotherapy	144 (95.4%)
Second‐line immunotherapy	39 (25.8%)
ICU	27 (17.9%)
mRS (3–6)	
At peak	116 (76.8%)
At discharge	35 (23.2%)
Tumor	23 (15.2%)
Follow‐up duration, m	39.34 ± 24.97
Outcome at last follow‐up	
AEAE	26 (18.4%) (10 missed)
mRS (3–6)	12 (8.5%) (10 missed)
Relapse	75 (53.2%) (10 missed)

Abbreviations: AE, autoimmune Encephalitis; AEAE, autoimmune encephalitis‐associated epilepsy; EEG, electroencephalography; ICU, intensive care unit; m, month; mRS, modified Rankin Scale; y, year.

**FIGURE 2:**
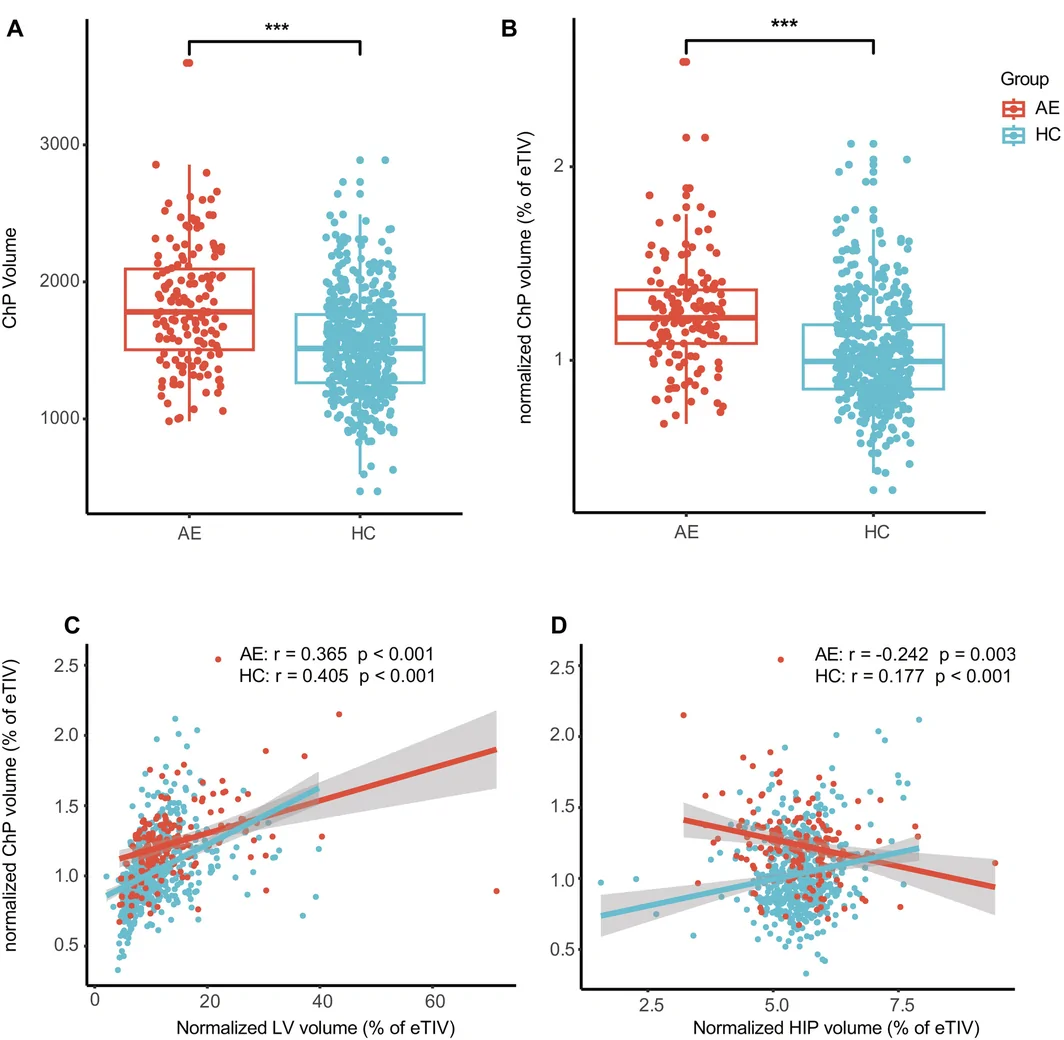
Volumetric assessment of the choroid plexus in autoimmune encephalitis. (A) Comparison of absolute choroid plexus (ChP) volume between patients with AE and healthy controls (HCs). (B) Comparison of normalized ChP volume (percentage of estimated total intracranial volume, eTIV). (C) Correlation between normalized ChP volume and normalized lateral ventricle volume (LV) in AE and HC groups. (D) Correlation between normalized ChP volume and normalized bilateral hippocampus (HIP) volume.

Compared with HCs, participants with AE displayed significantly larger CPV (1825.773 ± 439.005 mm^3^ vs. 1533.658 ± 361.301 mm^3^, *p* < 0.001), and this difference remained significant after normalization to eTIV (AE: 1.235 ± 0.274; HC: 1.035 ± 0.264, *p* < 0.001). Correlation analyses further revealed that normalized CPV was positively associated with normalized LVV in both groups; importantly, while a positive correlation was found between normalized CPV and normalized HIP volume in HCs, a negative correlation was observed in AE patients (Figure 2).

**FIGURE 3:**
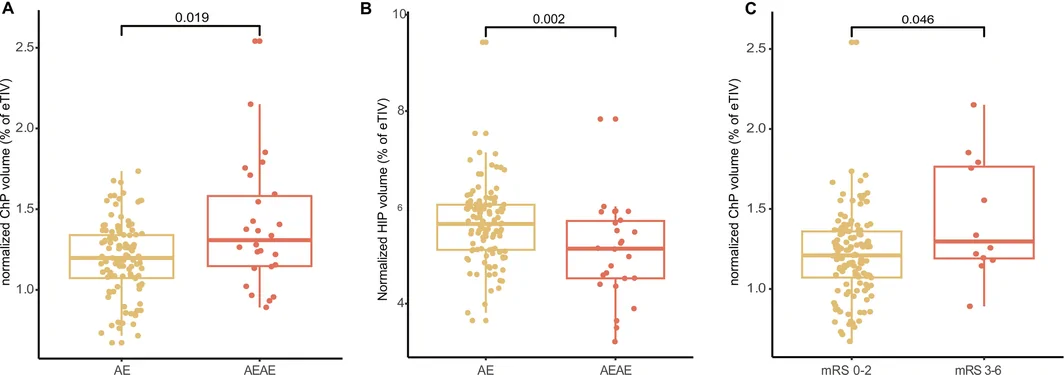
Volumetric comparisons of the choroid plexus and hippocampus in AE patients with different outcomes. (A) Comparison of normalized choroid plexus (ChP) volume between autoimmune encephalitis (AE) and those who developed autoimmune encephalitis‐associated epilepsy (AEAE). (B) Comparison of normalized hippocampus (HIP) volume between AE and AEAE groups. (C) Comparison of normalized ChP volume between patients with modified Rankin Scale (mRS) 0–2 and mRS 3–6.

To assess the prognostic value of these volumetric changes, we followed all AE patients for 1 year after excluding 10 lost‐to‐follow‐up cases. At baseline, the group that developed AEAE exhibited a larger normalized CPV but a smaller normalized HIP volume, and patients with mRS scores of 3–6 had a larger normalized CPV than those with scores of 0–2 (Figure 3). ROC analysis showed that using normalized CPV to predict AEAE yielded an AUC of 0.648 (95% CI: 0.554–0.742). The optimal cutoff value determined by the maximum Youden index was 1.36, with a sensitivity of 46.2% and a specificity of 79.1%. Mediation analysis showed that HIP volume did not mediate the association between ChP volume and chronic epilepsy (Figure S1).

**FIGURE 4:**
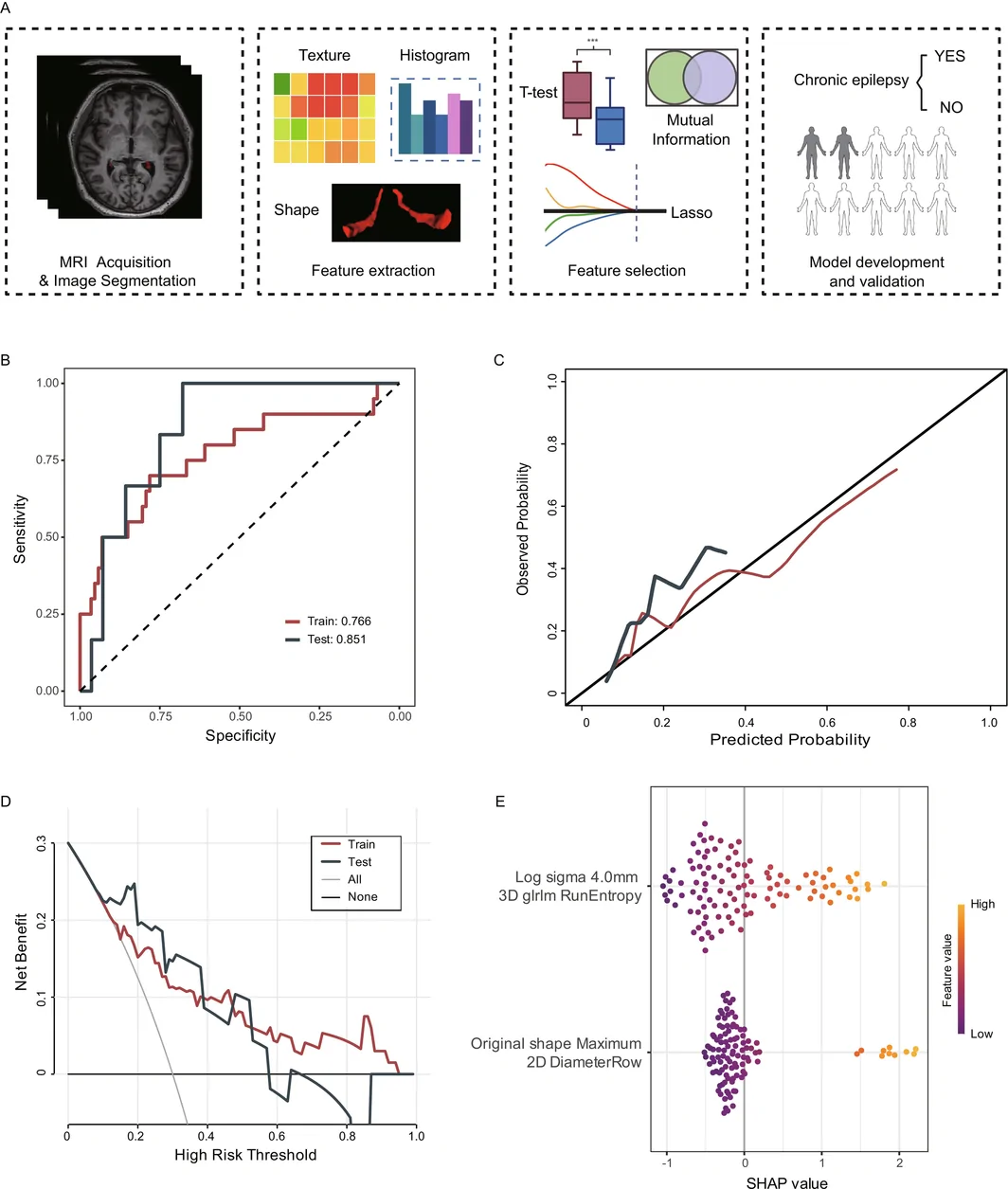
Radiomics‐enabled machine learning prediction of autoimmune encephalitis with chronic epilepsy using MRI features. (A) Workflow of radiomics‐based machine learning prediction for autoimmune encephalitis‐associated epilepsy (AEAE). (B) Receiver operating characteristic (ROC) curves for the training and test sets. (C) Calibration curve of the prediction model. (D) Decision curve analysis (DCA) for the model. (E) SHAP (SHapley Additive exPlanations) plot showing feature importance.

Further, from 944 radiomic features, we selected two representative features (RunEntropy and original volume) for a logistic regression model, which performed robustly, with an AUC of 0.766 in the training set and 0.851 in the test set, and accuracy, sensitivity, and specificity at optimal thresholds of 0.78, 0.80, and 0.76, respectively; ROC, calibration, and DCA confirmed strong performance. SHAP analysis highlighted these two features as key drivers, revealing that macroscopic geometry and microscopic texture heterogeneity underpin accurate modeling (Figure 4). Multivariable logistic regression confirmed that the radiomics‐defined high‐risk status remained an independent predictor of AEAE after adjusting for potential confounders (*p* = 0.002; Table 2).

**TABLE 2:** Multivariable logistic regression model for predicting autoimmune encephalitis‐associated epilepsy.

Variable	β coefficient	OR (95% CI)	*p*
Age	0.027	1.028 (0.979–1.079)	0.275
Gender	0.937	2.553 (0.788–8.273)	0.118
Subtypes (Ref: GABAB‐AE)			
GAD65‐AE	1.222	3.394 (0.395–29.161)	0.265
LGI1‐AE	−1.303	0.272 (0.059–1.258)	0.096
NMDA‐AE	−1.284	0.277 (0.031–2.481)	0.251
Prodromal symptoms	−0.401	0.670 (0.192–2.333)	0.529
Radiomics high‐risk	1.961	7.103 (2.089–24.146)	0.002
Corticosteroids therapy	−0.213	0.808 (0.202–3.234)	0.764
Second‐line immunotherapy	1.707	5.514 (1.553–19.574)	0.008
Intercept	−4.726	NA	NA

*Note:* Radiomics high‐risk was defined as a Rad‐score above the optimal cutoff determined by the maximum Youden index.

Abbreviations: AE, autoimmune encephalitis; GABAB‐AE, gamma‐aminobutyric acid type B receptor encephalitis; GAD65‐AE, glutamic acid decarboxylase 65 encephalitis; LGI1‐AE, leucine‐rich glioma‐inactivated protein 1 encephalitis; NA, not applicable; NMDAR‐AE, N‐methyl‐D‐aspartate receptor encephalitis.

**FIGURE 5:**
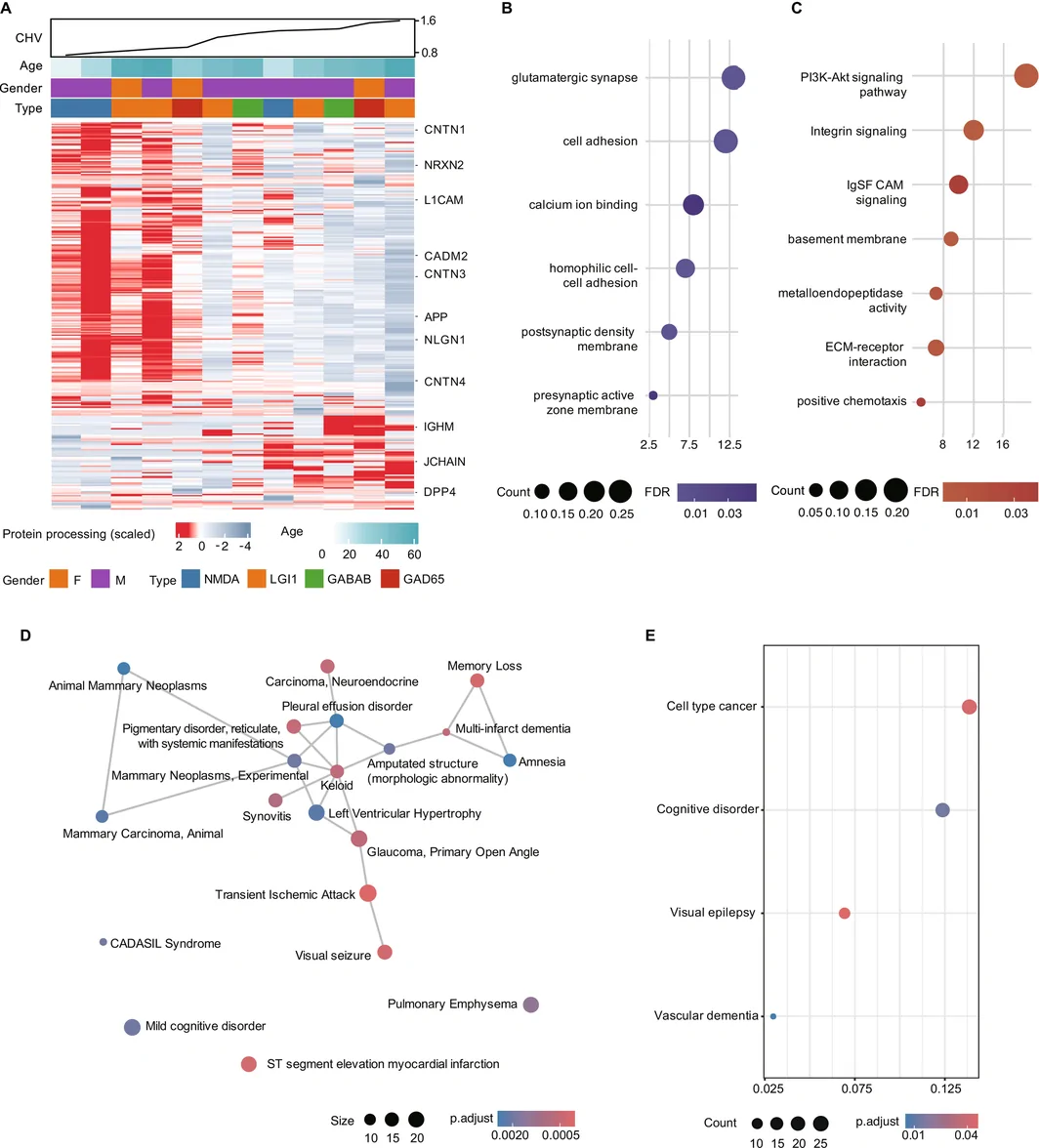
Proteomic profiling of proteins correlated with choroid plexus volume. (A) Heatmap displaying proteins significantly correlated with choroid plexus volume (CPV), annotated with clinical variables including age, sex, and AE subtype. (B) Enriched biological processes among proteins negatively correlated with CPV. (C) Enriched biological processes among proteins positively correlated with CPV. (D) Disease Ontology (DO) enrichment network of CPV‐correlated proteins. (E) DO enrichment results for specific diseases.

**FIGURE 6:**
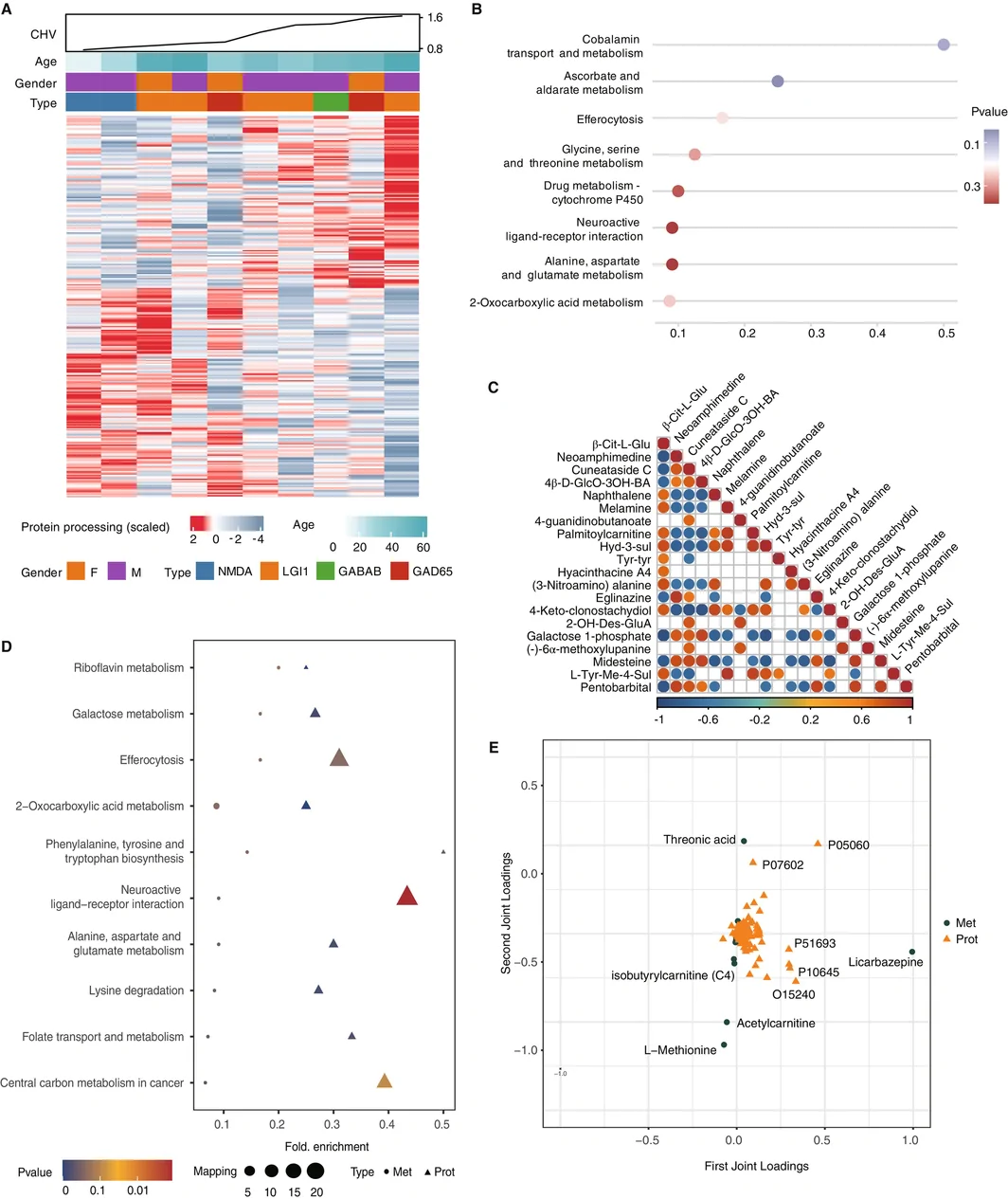
Metabolomic profiling of metabolites correlated with choroid plexus volume. (A) Heatmap displaying metabolites significantly correlated with choroid plexus volume (CPV), annotated with clinical variables including age, sex, and AE subtype. (B) Significantly enriched metabolic pathways among CPV‐correlated metabolites. (C) Correlation heatmap depicting pairwise correlations among CPV‐associated metabolites. (D) Joint enrichment analysis of metabolic pathways derived from both CPV‐correlated proteins and CPV‐correlated metabolites. (E) Joint loading plot of metabolites (Met) and proteins (Prot) from the integrative multivariate model.

To explore the molecular underpinnings of ChP enlargement, we performed exploratory proteomic and metabolomic profiling in a small CSF subset. Proteomic analysis revealed distinct patterns of CPV‐correlated proteins (Figure 5A). Functional enrichment analysis showed that proteins negatively correlated with CPV were enriched in glutamatergic synapse and cell adhesion pathways, while positively correlated proteins were enriched in PI3K‐Akt signaling, integrin signaling, ECM‐receptor interaction, and positive chemotaxis pathways (Figure 5B,C). Disease Ontology enrichment analysis further indicated potential associations with cognitive disorders, visual epilepsy, and vascular dementia (Figure 5D,E). Of the 204 ChP volume‐associated proteins, 159 (77.9%) showed reliable RNA expression in choroid plexus tissue, and high‐confidence STRING analysis identified seven that directly interact with blood–CSF barrier core proteins TJP1 and LAMB1 (Figure S2). Metabolomic profiling similarly showed distinct abundance patterns of ChP volume‐correlated metabolites (Figure 6A); with enrichment analysis pointing to perturbations in neuroactive ligand‐receptor interaction pathways (Figure 6B). Correlation analysis depicted coordinated alterations among key metabolites (Figure 6C). Integrated multi‐omics analysis illustrated potential crosstalk between metabolites and proteins involved in synaptic and metabolic pathways (Figure 6D,E).

## 4 Discussion

Our results show that choroid plexus volume (CPV) is significantly enlarged in AE patients compared with HCs, a finding consistent with previous reports in other neuroinflammatory and neurodegenerative conditions [32, 33, 34]. Notably, among AE patients, a larger baseline CPV was associated with the subsequent development of autoimmune encephalitis‐associated epilepsy (AEAE) and with poorer functional outcomes. Moreover, ChP‐derived radiomic features independently predicted AEAE. These findings indicate that ChP enlargement is a feature of AE and that ChP measures may serve as early predictors of subsequent AEAE and worse functional outcomes.

The contrasting ChP‐HIP volume correlations in AE versus HCs are particularly noteworthy. The shift from a positive correlation in healthy individuals to a negative one in AE patients suggests a fundamental disease‐related change in this dynamic interplay. Notably, our mediation analysis showed that the predictive value of CPV for chronic epilepsy was not mediated by hippocampal atrophy, pointing to parallel or independent pathological pathways. ChP dysfunction may therefore contribute to epileptogenesis through mechanisms distinct from hippocampal damage, such as altered CSF composition or secretion of pro‐inflammatory factors that directly affect neuronal excitability [35].

Our univariate analysis identified an optimal cutoff for normalized CPV in predicting AEAE; however, the volume‐based model showed only modest performance and is not suitable for standalone decision‐making. In contrast, our radiomic model confirmed that the ChP's predictive value extends beyond simple volumetry. Shape‐based and textural features captured microarchitectural alterations—such as changes in vascularity, cellular remodeling, and stromal composition—that conventional measures miss. SHAP analysis further identified CPV as an important contributor to the radiomic signature, consistent with our univariate findings and with a recent study in MS and neuromyelitis optica spectrum disorder [36]. Collectively, these results support the potential of ChP radiomics as a biomarker in neuroinflammatory research.

Observed ChP enlargement in AE might reflect pathological remodeling (e.g., immune infiltration, vascular proliferation, epithelial activation/edema), tentatively supported by our proteomic data showing CNS inflammation with immunoglobulin‐mediated responses. Inflammation‐induced BCSFB hyperpermeability, previously implicated in AE [37], could facilitate autoantibody entry and formation [38]. Viral infections may promote cross‐reactive antibodies and proinflammatory cytokines (e.g., IL‐17) that damage barrier components [38, 39, 40]; proinflammatory states might also prime microglia to release mediators compromising barrier integrity, potentially perpetuating neuroinflammation [41, 42, 43]. Our exploratory proteomics, based on a small subset of patients, suggested that larger CPV was associated with downregulation of glutamatergic synapse pathways and upregulation of PI3K‐Akt, integrin, and ECM‐receptor pathways. The downregulation of glutamatergic signaling might hypothetically contribute to epileptogenesis in AEAE, while the upregulation of integrin and chemotaxis pathways is consistent with a potential role for the enlarged ChP in promoting immune cell trafficking across the BCSFB [44]. Previous work links ventricular inflammation to ChP‐derived growth factors and epithelial expansion [45], providing a possible upstream mechanism. Together, these exploratory findings raise the possibility that the ChP could promote epileptogenesis via inflammatory milieu and neuroactive molecules, but given the small sample size, all pathway‐level results are hypothesis‐generating and need validation in larger cohorts.

Regrettably, we cannot fully determine whether ChP enlargement is a cause or consequence of neuroinflammation—a limitation shared with other neuroinflammatory and neurodegenerative disorders including MS, AD, and Parkinson's disease (PD) [34, 46, 47, 48, 49]. Two plausible and non‐exclusive mechanisms may underpin this association: persistent peripheral immune activation during the post‐acute phase may induce ChP injury, stromal fibrosis, and structural disruption leading to enlargement, supported by evidence in MS where CPV is a neuroinflammatory biomarker [50, 51]. Conversely, an enlarged ChP may increase blood‐CSF barrier permeability, facilitating immune cell and cytokine infiltration and thereby perpetuating neuroinflammation. Longitudinal prospective studies incorporating serial imaging and inflammatory profiling are urgently needed to clarify causality and validate ChP volumetry as a reliable imaging biomarker in AE.

This study has several limitations. First, despite a relatively large AE cohort, the single‐center design and the very small sample sizes for proteomic/metabolomic profiling constrain generalizability. Second, our mechanistic inferences are purely correlational—animal models and in vivo molecular imaging are essential to verify causal relationships between ChP alterations and AE pathology. Third, the cross‐sectional design precludes delineation of the temporal sequence and causal link between ChP morphological dynamics and neuroinflammatory activation.

## 5 Conclusion

In conclusion, our study demonstrates that enlarged ChP volume is a prominent imaging feature in patients with AE, and this morphological change is associated with poor prognosis. Future studies with larger, multicenter cohorts and in‐depth mechanistic validation are warranted to further confirm the clinical utility of ChP in AE.

## Author Contributions

Q.W. and Y.S. conceived, designed, and supervised the study. Y.S., N.W., and S.G. acquired the data. Y.S. analyzed and interpreted the data, provided statistical analysis, had full access to all of the data in the study, and is responsible for the integrity of the data and the accuracy of the data analysis. Y.S. drafted the manuscript. Q.W. and L.F. critically revised the manuscript for important intellectual content. All authors read and approved the final manuscript.

## Funding

The study was financially supported by the National Natural Science Foundation of China (82501734, 82371449), the Capital Health Research and Development of Special grants (2024‐1‐2041), the National Key R&D Program of China grant (2022YFC2503800), the China Postdoctoral Science Foundation (2024M762180), Beijing Postdoctoral Research Foundation, CAAE Epilepsy Care Fund (CK‐2025‐020).

## Ethics Statement

This study was approved by the Institutional Review Board of Capital Medical University, Beijing Tiantan Hospital (Beijing, China) (KY2023‐079‐01).

## Consent

All participants or their caregivers provided written informed consent for the publication.

## Conflicts of Interest

The authors declare no conflicts of interest.

## Supporting information


**Figure S1:** Mediation analysis of the relationship between choroid plexus, hippocampus, and autoimmune encephalitis‐associated epilepsy. (A) Mediation analysis results showing average causal mediation effect (ACME), average direct effect (ADE), and total effect. (B) Mediation model depicting the relationship between choroid plexus (ChP), hippocampus (HIP), and autoimmune encephalitis‐associated epilepsy (AEAE), with ACME, proportion mediated (Prop. Mediated), and ADE presented.

**Figure S2:** RNA expression validation and high‐confidence protein–protein interaction (PPI) network of choroid plexus differentially expressed proteins (DEPs). (A) Pie chart showing the expression ratio of 204 choroid plexus‐related DEPs based on Human Protein Atlas RNA‐seq data; 77.9% of DEPs are detectable in choroid plexus (nTPM ≥ 1). (B) STRING PPI network (combined score ≥ 0.7): seven DEPs (light blue) interact with core blood–CSF barrier proteins TJP1 and LAMB1 (red), while CDH2 (orange) acts as an adherens junction component. Edge properties indicate interaction confidence. Abbreviations: nTPM, normalized transcripts per million.

## Data Availability

The data that support the findings of this study are available from the corresponding author upon reasonable request.
